# Investigating the Efficacy of Triple Artemisinin-Based Combination Therapies for Treating Plasmodium falciparum Malaria Patients Using Mathematical Modeling

**DOI:** 10.1128/AAC.01068-18

**Published:** 2018-10-24

**Authors:** Saber Dini, Sophie Zaloumis, Pengxing Cao, Ric N. Price, Freya J. I. Fowkes, Rob W. van der Pluijm, James M. McCaw, Julie A. Simpson

**Affiliations:** a Centre for Epidemiology and Biostatistics, Melbourne School of Population and Global Health, University of Melbourne, Melbourne, Australia; b School of Mathematics and Statistics, University of Melbourne, Melbourne, Australia; c Global and Tropical Health Division, Menzies School of Health Research and Charles Darwin University, Casuarina, Australia; d Centre for Tropical Medicine and Global Health, Nuffield Department of Clinical Medicine, University of Oxford, Oxford, United Kingdom; e Burnet Institute, Disease Elimination Program, Public Health, Melbourne, Australia; f Department of Epidemiology and Preventative Medicine and Department of Infectious Diseases, Monash University, Melbourne, Australia; g Mahidol Oxford Tropical Medicine Research Unit, Bangkok, Thailand; h Peter Doherty Institute for Infection and Immunity, The Royal Melbourne Hospital and University of Melbourne, Melbourne, Australia; i Murdoch Children's Research Institute, The Royal Children's Hospital, Melbourne, Australia

**Keywords:** antimalarial agents, artemisinin-based combination therapy, drug efficacy, drugs interactions, mathematical modeling

## Abstract

The first line treatment for uncomplicated falciparum malaria is artemisinin-based combination therapy (ACT), which consists of an artemisinin derivative coadministered with a longer-acting partner drug. However, the spread of Plasmodium falciparum resistant to both artemisinin and its partner drugs poses a major global threat to malaria control activities. Novel strategies are needed to retard and reverse the spread of these resistant parasites. One such strategy is triple artemisinin-based combination therapy (TACT). We developed a mechanistic within-host mathematical model to investigate the efficacy of a TACT (dihydroartemisinin-piperaquine-mefloquine [DHA-PPQ-MQ]) for use in South-East Asia, where DHA and PPQ resistance are now increasingly prevalent. Comprehensive model simulations were used to explore the degree to which the underlying resistance influences the parasitological outcomes. The effect of MQ dosing on the efficacy of TACT was quantified at various degrees of DHA and PPQ resistance. To incorporate interactions between drugs, a novel model is presented for the combined effect of DHA-PPQ-MQ, which illustrates how the interactions can influence treatment efficacy. When combined with a standard regimen of DHA and PPQ, the administration of three 6.7-mg/kg doses of MQ was sufficient to achieve parasitological efficacy greater than that currently recommended by World Health Organization (WHO) guidelines. As a result, three 8.3-mg/kg doses of MQ, the current WHO-recommended dosing regimen for MQ, combined with DHA-PPQ, has the potential to produce high cure rates in regions where resistance to DHA-PPQ has emerged.

## Abstract (precis)

The first line treatment for uncomplicated falciparum malaria is artemisinin-based combination therapy (ACT), which consists of an artemisinin derivative coadministered with a longer-acting partner drug. However, the spread of Plasmodium falciparum resistant to both artemisinin and its partner drugs poses a major global threat to malaria control activities.

## INTRODUCTION

Over the last decade, significant gains have been made towards the control and elimination of malaria. Despite this progress, almost half a million people still die from malaria each year. Disturbingly, in 2016 there were five million more cases of malaria than the previous year (according to the World Health Organization [WHO] in 2017 [1]), emphasizing the fragile nature of malaria control. Early diagnosis and treatment with highly effective antimalarial drug regimens remains central to all national malaria control activities. Artemisinin-based combination therapies (ACTs) are the first-line therapy in almost all countries where malaria is endemic due to the high efficacy, tolerability, and ability of ACTs to reduce ongoing transmission of the parasite. ACTs are comprised of two components: an artemisinin derivative and a partner drug. The artemisinin derivative has a high antimalarial potency, killing a large proportion of parasites; however, these compounds are rapidly eliminated, leaving a residual parasite population that, if left untreated, will likely recrudesce. A slowly eliminated partner drug is required to provide a sustained antimalarial activity that is capable of killing the remaining parasites (2).

ACTs have remained highly efficacious for almost 2 decades but are now under threat from the emergence of drug-resistant parasites (2, 3). In 2009, a high proportion of patients with markedly delayed parasite clearance were reported from the western region of Cambodia, and this was confirmed as being attributable to artemisinin resistance (3). These parasites have now spread across the Greater Mekong Region (4, 5). Delayed parasite clearance and higher gametocyte carriage, due to the artemisinin derivative resistance, drive the selection of resistance to the partner drug (6), and in South-East Asia this has resulted in declining efficacy of all the ACTs currently recommended by WHO (7). In some parts of the Greater Mekong Region, the spread of highly drug-resistant parasites poses a major threat to malaria control activities. The emergence of an untreatable P. falciparum will result in an inevitable rise in malaria incidence, epidemics, and associated morbidity and mortality.

The development of alternative strategies is crucial to ensuring the ongoing success of malaria control efforts. Triple Artemisinin-based Combination Therapy (TACT) is a novel strategy by which a new partner drug is added to an established ACT. TACT has the potential to decrease the chance of emergence of a *de novo* resistance, as well as rescuing a regimen in which one of the ACT components is already failing. Two antimalarial clinical trials are under way to determine the efficacy of TACT for uncomplicated falciparum malaria: Artemether-Lumefantrine plus Amodiaquine (AL-AQ) and Dihydroartemisinin-Piperaquine plus Mefloquine (DHA-PPQ-MQ). These are being compared to the standard ACTs (AL and DHA-PPQ) alone (see trial NCT02453308 at clinicaltrials.gov).

In the present study, we developed a within-host mathematical model (8) to explore the efficacy of TACTs, with a particular focus on DHA-PPQ-MQ, since DHA-PPQ is widely administered in South-East Asia and is currently associated with very high failure rates in some regions (9–11). The model accommodates a high level of biological details, such as drug-drug interaction (12, 13), the stage specificity of parasite killing (14–17), and between-patient and between-isolate variability (17). We used the model to simulate different levels of resistance and quantify the degree to which this compromises the efficacy of TACT. The optimal MQ dosing regimen was determined for various degrees of resistance to DHA-PPQ.

## RESULTS

**FIG 1:**
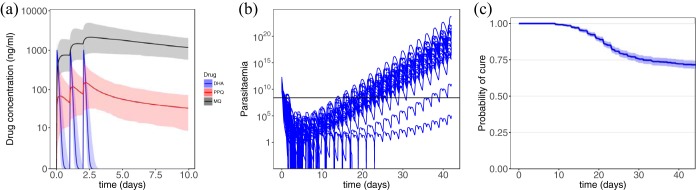
Model simulation. (a) PK model results. The concentrations of DHA (blue), PPQ (red), and MQ (black) are depicted. The shaded regions show the area between the 2.5th and 97.5th percentiles of the results generated for 1,000 patients. (b) PD model results for 100 randomly selected patients. The horizontal line shows the microscopic level of detection of parasites. (c) Kaplan-Meier estimation of the probability of survival over 42 days of follow-up.

Figure 1 shows examples of drug concentration profiles for DHA, PPQ, and MQ and the corresponding parasitemia profiles obtained from model simulations. WHO-recommended dosing regimens were used in the simulations, i.e., 18.0 mg/kg/day of PPQ, 4.0 mg/kg/day of DHA, and 8.3 mg/kg/day of MQ for 3 days. The median concentrations of the drugs (lines), along with the between-subject variabilities (the shaded regions show the area between the 2.5 and 97.5% percentiles), are presented in Fig. 1a, and the parasitemia of 100 randomly selected patients in Fig. 1b. Figure 1c presents the Kaplan-Meier estimation of the probability of cure, along with the 95% confidence intervals illustrated by the shaded region.

**FIG 2:**
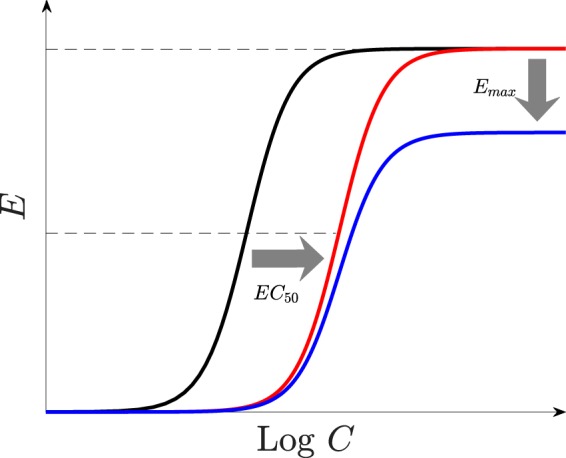
Resistance manifestations. The graph shows the resistance of parasites to drugs, modeled by relevant alterations of the parameters of the model. A concentration-effect profile of susceptible parasites (black) can be right-shifted, i.e., the EC_50_ increases (red) and/or the maximum killing effect, *E*_max_, decreases (blue).

Parasite resistance to antimalarial drugs can manifest in a couple of different ways that affect the killing profile of a drug (see the concentration-effect curves in Fig. 2). These include (i) increasing the 50% effective concentration (EC_50_; the red curve), (ii) reducing the size of the killing window in the intraerythrocytic parasite life cycle, *W*, and (iii) decreasing the sigmoicity, γ, and/or maximum killing effect, *E*_max_ (the blue curve). The degree of resistance was modeled initially by varying the EC_50_ values of PPQ, in scenarios where the parasites are sensitive or resistant to DHA. The influence of other manifestations of resistance on TACT efficacy are outlined in the supplemental material.

The level of resistance and the resultant risk of treatment failure varies with geographical region. Table 1 demonstrates a large variation in DHA-PPQ efficacy in different regions across South-East Asia (18). The risk of failures in Aoral and Chi Kraeng in Cambodia are 51.9 and 62.5% treatment failures, respectively, whereas in Siem Pang it is only 8.3%. Similar large variations in the probability of treatment failure are observed in Vietnam. According to the WHO treatment guidelines, when the risk of failure exceeds 10%, a treatment is considered suboptimal, and steps should be taken to change the policy to a more efficacious antimalarial regimen.

**TABLE 1:** Kaplan-Meier estimation of the probabilities of cure on day 42 of follow-up in some regions in South-East Asia where DHA-PPQ is the first-line treatment for malaria^*a*^

Parameter	Geographical region				
	Siem Pang	Binh Phuoc	Bu Gia Map	Aoral	Chi Kraeng
Probability of cure	0.92	0.74	0.67	0.48	0.38
No. of patients	60	40	40	53	40
Yr	2015	2015	2015	2015	2014
Country	Cambodia	Viet Nam	Viet Nam	Cambodia	Cambodia

a World Health Organization (18).

### Artemisinin sensitivity.

**FIG 3:**
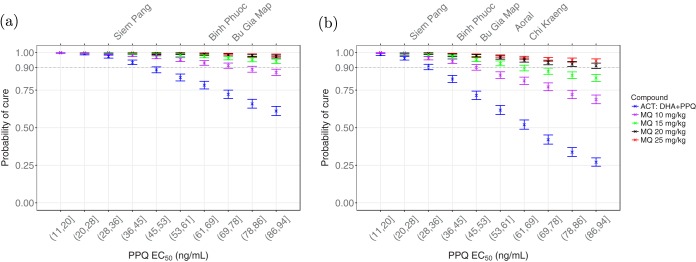
The probability of cure on day 42 of follow-up when EC_50_ of PPQ varies over the deciles of [11 94]. (a and b) Sensitivity (a) and resistance (b) to DHA. Blue, ACT treatment (the dosing regimens of PPQ and DHA are 18.0 mg/kg and 4.0 mg/kg, respectively, on days 1, 2 and 3); purple, a 10-mg/kg (3.3 mg/kg/day for 3 days) dose of MQ is added; green, a 15-mg/kg (5 mg/kg/day for 3 days) dose of MQ is added; black, a 20-mg/kg (6.7 mg/kg/day for 3 days) dose of MQ is added; red, a 25-mg/kg (8.3 mg/kg/day for 3 days) dose of MQ is added. The top labels show the geographical regions in South-East Asia (Table 1) where DHA-PPQ cure rates equal to the corresponding simulated values have been observed. Error bars show the 95% confidence intervals from Kaplan-Meier analysis.

In the first investigated scenario, the parasites were assumed to be sensitive to DHA (the sampling interval of EC_50,*D*_ was limited to (1,10] ng/ml), and the resistance level to PPQ was varied. Figure 3a shows how the probability of cure at day 42 of follow-up varies with EC_50_ of PPQ over the deciles of (11,94] ng/ml. The top labels in this figure show the geographical regions in South-East Asia (Table 1) that have observed DHA-PPQ day 42 cure rates equal to the corresponding simulated values (18).

The probability of cure declines as the EC_50_ of PPQ increases. Without MQ, the probability of cure with DHA-PPQ is below 90%, over EC_50,*P*_ ∈ (45,94], which includes Binh Phuoc and Bu Gia Map. This scenario was unable to produce the probabilities of cure observed in all of the geographical regions, shown in Table 1.

The addition of a 10-mg/kg total dose of MQ (3.3 mg/kg/day for 3 days) significantly raised the probability of cure. The improvement in efficacy with this regimen of MQ was insufficient to ensure successful treatment in Bu Gia Map. In this region, 15-mg/kg dose of MQ (5 mg/kg/day for 3 days) was required. When the parasites are sensitive to DHA but resistant to PPQ, MQ at 15 mg/kg was sufficient to achieve cure in all locations. Administration of 20 mg/kg (6.7 mg/kg/day for 3 days) or 25 mg/kg (8.3 mg/kg/day for 3 days) did not provide significant benefit over the 15-mg/kg regimen, although this might be used to guarantee the success of the TACT.

### Artemisinin resistance.

Concurrent resistance to DHA and PPQ is now documented in Cambodia and Vietnam (9, 10). To simulate a high level of DHA resistance, we set EC_50,*D*_ ∈ (50,100] ng/ml and varied the intensity of resistance to PPQ, EC_50,*P*_ ∈ (11,94] ng/ml. Using the same dosing regimens as those in Fig. 3a, resistance to DHA leads to a significant decline in the efficacy of DHA-PPQ, as shown in Fig. 3b. When combined with a 10-mg/kg dose of MQ, the efficacy of the TACT was improved significantly, but except for Siem Pang it was clearly not sufficient.

Administration of a 15-mg/kg dose of MQ provided sufficient efficacy in Binh Phuoc and Bu Gia Map but was still insufficient for Aoral and Chi Kraeng. At least a 20-mg/kg dose of MQ was needed to obtain a successful treatment in all of the regions. Of note, administering 25 mg/kg of MQ (8.3 mg/kg/day for 3 days), which is currently the recommended dosing regimen by the WHO for the ACT of MQ plus artesunate (19), can produce probability of cure above 90% at all of the DHA-PPQ resistance levels.

### Influence of the antagonistic PPQ-MQ interaction.

**FIG 4:**
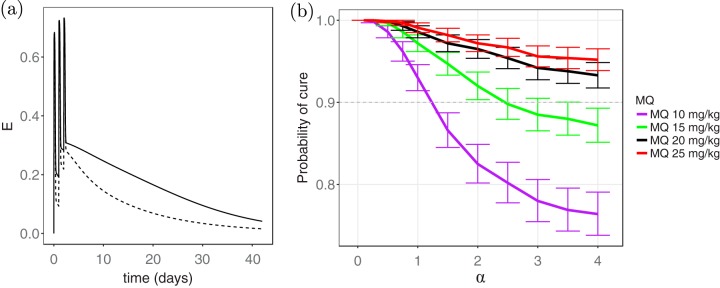
Influence of antagonism between PPQ and MQ on the efficacy of the TACT. (a) Dashed and solid lines represent combined killing effect, *E*, for α = 3.3 and α = 1, respectively. (b) Probability of cure on day 42 of follow-up versus the interaction parameter, α, when the resistance level corresponding to Chi Kraeng is considered, i.e., EC_50_ ∈ (69, 78]; resistance to DHA is assumed. Different values of the interaction parameter, α, produce synergism (0 < α < 1), zero interaction (α = 1), and antagonism (0 < α < ∞) in the combined effect of PPQ-MQ. The interpretation of the colors is explained in the caption to Fig. 3.

The effect of the PPQ-MQ interaction parameter, α, on the probability of cure was then investigated. Figure 4a shows the combined killing effect of the drugs, *E*, over time for a selected patient with two different values of the interaction parameter: α = 3.3 (antagonism) and α = 1 (zero interaction); the other parameters were kept constant. The killing effect for α = 1 (solid line) is significantly higher than that for α = 3.3 (dashed line), indicating the extent to which the drugs can nullify each other's effect and the loss in the overall efficacy of TACT.

The effect of the interaction parameter, α, on the efficacy was further assessed by restricting the resistance level to that corresponding to Chi Kraeng (EC_50,*P*_ ∈ (69,78]), for instance, and estimating the probability of cure for different values of α; DHA resistance is also assumed. The results demonstrated a significant difference between the probabilities of cure at different values of α (Fig. 4b), for example, when α < 1 (synergism), adding a 10-mg/kg dose of MQ was enough to provide 90% efficacy. In contrast, when α > 1 (antagonism), the probability of cure fell well below 90%. Similarly, the probability of cure declined with increasing α (i.e., antagonism intensification) for MQ administration with higher doses. Of note, 20- and 25-mg/kg dosing regimens of MQ achieved greater than 90% efficacy at all values of α, even at levels indicative of very strong antagonism. This highlights the robustness of these dosing regimens in producing a successful treatment. The antagonism between PPQ and MQ had an important impact on the efficacy of the TACT, and neglecting this may result in an underestimation of the dose of MQ required for successful treatment across different regions.

The effect of other manifestations of resistance on the efficacy of the TACT are illustrated in the supplemental material. Figure S1 presents the probability of survival at different levels of resistance produced by varying the maximum killing effect of PPQ, *E*_max,*P*_. Similar to the case where EC_50_ was the manifestation of resistance, shown in Fig. 3, the results indicate that the three 6.7- or 8.3-mg/kg doses of MQ are sufficient to provide the desirable probability of cure at every level of resistance. The outcomes were consistent when the killing window of PPQ was shortened, as shown in Fig. S2 in the supplemental material. However, the probability of cure became extremely low, to an extent that the efficacy of 20-mg/kg dosing regimen fell below 90% at very high levels of resistance, although observing such high resistance levels is currently unlikely. Nevertheless, a 25-mg/kg dosing regimen of MQ overcame such high levels of resistance and ensured high probability of cure.

## DISCUSSION

We have presented a novel mathematical model to investigate the efficacy of different regimens for triple artemisinin combination therapies (TACTs). Our analysis focused on DHA-PPQ-MQ, since DHA-PPQ is a widely used ACT in South-East Asia with declining efficacy in several locations (9–11). The addition of MQ to DHA-PPQ has potential to improve treatment, since the ACT of artesunate-MQ retains high efficacy, following its reintroduction as a first-line treatment in Cambodia (7). Our results suggest that a 10-mg/kg (3.3 mg/kg/day for 3 days) dose of MQ can improve the treatment efficacy of DHA-PPQ significantly and would be an appropriate regimen for regions such as Siem Pang in Cambodia and Binh Phuoc in Viet Nam. However, it is likely to be insufficient in regions where there is preexisting high-grade resistance to PPQ, such as Bu Gia Map in Viet Nam and Aoral and Chi Kraeng in Cambodia. Administration of DHA-PPQ with 15 mg/kg (3 mg/kg/day for 3 days) of MQ would be beneficial, but efficacy would still be compromised in the regions where there was high level of resistance to both PPQ and DHA, such as Chi Kraeng. To achieve a cure rate of greater than 90%, as recommended by the WHO, at least, 20 mg/kg (6.7 mg/kg/day for 3 days) of MQ needs to be administered in conjunction with the standard 3-day regimen of DHA-PPQ. As a result, the WHO-recommended dosing regimen of MQ, i.e., 25 mg/kg (8.3 mg/kg/day for 3 days), which has already been shown to be well tolerated and safe, has high potential to provide cure rates above 90%.

Our model enabled us to simulate the pharmacokinetics (PK) and pharmacodynamics (PD) following TACT administration to patients with malaria and provided important insights into the way in which the underlying mechanisms of drug action affect treatment efficacy. By taking account of between-patient and between-isolate variability, we were able to explore treatment efficacy across a wide range of different scenarios reflecting various parasite resistances to the different drug components. The results showed similar trends for different resistance manifestations, confirming the robustness of the proposed dosing regimen of DHA-PPQ-MQ.

We have proposed a novel empirical model to accommodate the effect of the combined drugs, assuming that PPQ and MQ (both quinoline compounds) have similar modes of action, which differs from that of DHA (an endoperoxide compound). The killing effects of PPQ-MQ and DHA were therefore assumed to be independent. This justified using a combination of Bliss independence and Loewe additivity to define the combined effect of the whole compound (see equation 1).

To facilitate the dissemination of our model and assist clinical researchers to investigate how different PK and PD parameters and dosing schemes influence parasitological outcomes, we produced an online application (appTACT [http://lab.qmalaria.org/shiny/appTACT/]) that allows varying the values of parameters and simulating the model.

Our mathematical model can be used to guide the development of suitable TACT regimens for investigation in clinical trials. Determining dosing regimens that are robust to a wide range of scenarios helps rationalize the logistical and financial challenges of phase 2 and 3 clinical trials. Further improvements in the model can be made to increase its fidelity to the underlying biology, for instance, by consideration of the artemisinins PK (e.g., bioavailability) dependence on parasite density (20) and different bioavailabilities of MQ at different administered days (21). The PD model can also be improved by incorporating more complexities underlying drug action, such as the dependence of killing effect on the timespan that parasites are exposed to drugs (22–25), immunity-mediated parasite killing (26, 27), and red blood cell (RBC) depletion/production (28, 29). However, in this initial analysis, we aimed to focus on the generality of the model and study worst-case scenarios, e.g., insignificant acquired immunity (patients living in low-endemicity regions), and negligible increased sensitivity of parasites to MQ in some regions, associated with PPQ resistance (10, 30). Although we did not explore the degree to which the efficacy of TACT influenced other aspects of malaria control, such as the transmissibility of the parasite, this certainly warrants further investigation, since a more comprehensive perspective will be needed on the suitability of deploying TACT in areas of high drug resistance.

## MATERIALS AND METHODS

### Mathematical Model.

The pharmacokinetic-pharmacodynamic (PK-PD) model presented in Zaloumis et al. (17) was used to model the dynamics of drug concentrations and parasite burden within an individual. In brief, this model describes the time-evolution of the number of parasites in the body, *N*, by the following difference equations:

$$N(a,t)=\{\begin{array}{ll} N\left(a-1,t-1\right)(1-E\left(a-1,t-1)\right), & 1<a\leq 48,\\ N\left(48,t-1\right)(1-E\left(48,t-1)\right)\times \text{PMF}, & \;a=1, \end{array}$$

where *a* is the parasites' age, taking only integer values over [1 48], *t* is time, and PMF is the parasite multiplication factor, which represents the number of merozoites released into blood by a shizont at the end of its life cycle. *E*(*a*, *t*) is the combined killing effect of the drugs and has been modified from that presented in Zaloumis et al. (17) to account for three drugs and accommodate drug interactions. The combined killing effect of the drugs is between 0 and 1 and is dependent upon the age of parasites during (*t*, *t* + 1). The number of detectable parasites circulating in the blood, *M*(*t*), is determined as follows:

$$M\left(t\right)=\overset{48}{\underset{a=1}{\sum }}N\left(a,t\right)g\left(a\right),$$

where *g*(*a*) accounts for the reduction in the number of circulating parasites in the blood due to sequestration, estimated to be:

$$g\;(a)={\{}_{2^{\frac{11-a}{3},}}^{\begin{array}{c} 1, \end{array}}\begin{array}{c} \;a<11,\\ \;a\geq 11, \end{array}$$

where we assumed sequestration begins at age 11 and intensifies with age (16, 31). In the ensuing section, we explain the details of modeling the combined effect of the drugs, *E*.

Parasites can be cleared faster in patients who have acquired immunity to P. falciparum, although the effect is small relative to the effect of antimalarial drugs (32). Therefore, since a TACT regimen is sought that is as well effective in worst-case scenarios, e.g., in low-endemicity regions where resistance has been developed and acquired immunity within an individual is typically low, we did not incorporate the effect of immunity in the PD model. Red blood cell (RBC) depletion and production can also play important roles on the dynamics of malaria infection, as demonstrated earlier (28, 29). However, since the period between drug administration and the time to parasitemia recrudescence is of interest here, i.e., when RBC depletion is not yet significant, these factors are not incorporated in the model.

The PK models for the three drugs considered—DHA, PPQ, and MQ—are well characterized; one-compartment models were used for DHA and MQ, and a two-compartment model was used for PPQ (33–35). The PK parameter values are drawn from the literature and are provided in Table 2.

**TABLE 2:** Parameter values of the pharmacokinetic model^*a*^

PK parameter	Drug			Description
	DHA (34)	MQ (33)	PPQ (35)	
*k_a_* (1/h)	0.82 (26.5)	0.29 (26)	0.717 (168)	Absorption rate
CL/*F* (liters/kg/h)	1.01 (22.4)	0.03 (33)	1.38 (42)	Clearance
*V/F* (liters/kg)	0.83 (50)	10.2 (51)		Vol of distribution
*V_C_/F* (liters/kg)			180.42 (101)	Vol of central compartment
*Q*/*F* (liters/kg/h)			2.73 (85)	Intercompartmental clearance
*V_P_*/*F* (liters/kg)			500 (50)	Vol of peripheral compartment

a The mean values are shown, along with the between-patient variabilities (presented as the percent coefficient of variation [%COV]) in parentheses.

### Combined killing effect of the drugs.

The combined killing effect of the drugs is modulated by the manner in which they interact with each other. Synergistic interaction between drugs produces a stronger combined effect compared to the case where they do not interact, i.e., zero interaction (also known as pure additivity). Conversely, antagonistic drug-drug interactions can nullify their additive effect and produce a lower combined effect than that for the zero-interaction case. Therefore, to model the combined effect, *E*, we must first identify how the drugs interact.

An empirical approach was taken, modeling zero-interaction as the reference (null) model (36–38), since the mechanisms underlying the killing effects are complex and not completely understood (39). Among the existing empirical approaches of modeling zero-interaction, two are more prominent and widely used: Loewe additivity (40) and Bliss independence (41). Loewe additivity is suggested to be a suitable concept for zero-interaction when noninteracting drugs have similar modes of action, however, when the drugs are believed to act independently, Bliss independence is more appropriate (37, 38).

It has been suggested that MQ and PPQ kill parasites through a similar mechanism, involving the disruption of heme detoxification in the parasite vacuole (39, 42, 43). DHA has a different mode of action, which involves the generation of free radicals and reactive intermediates that target various proteins of parasites (42, 44, 45). The PK and PD interactions of DHA with PPQ and MQ appear to be negligible (13).

The independent mechanisms of action of DHA and PPQ-MQ justifies using the Bliss independence concept for modeling the combined killing effect, *E*, given by

(1) $$E=E_{D}+E_{PM}-E_{D}E_{PM},$$

where *E_D_* is the killing effect of DHA and *E_PM_* is the combined effect of PPQ and MQ.

We assume Michaelis-Menten kinetics for *E_D_*:

$$E_{D}=E_{\max\;,D}\frac{C_{D}^{\gamma_{D}}}{C_{D}^{\gamma_{D}}+EC_{50,D}^{\gamma_{D}}}1_{W_{D}}(a),$$

where *E*_max,*D*_ is the maximum killing effect of DHA, *C_D_* is the DHA concentration, *EC*_50,*D*_ is the concentration at which 50% of the maximum killing effect is obtained, γ_*D*_ is the sigmoidicity (also known as slope) of the concentration-effect curve, and **1**_*W*_(*a*) is an indicator function, used to implement the age-specific killing of drugs, defined by:

$$1_{W}\left(a\right)=\{\begin{array}{cc} 1, & a\in W,\\ 0, & \text{otherwise,} \end{array}$$

where *W* is the age window (interval) where the antimalarial drugs are able to kill the parasites and *W_D_* is the killing window of DHA.

To define *E_PM_*, models incorporating the Loewe additivity concept (as PPQ and MQ have similar modes of action) were used, which include only one parameter for the effect of the interaction between PPQ and MQ (37, 38, 46). These models are more specified to the framework of drug interaction, in contrast to the statistical models that usually have multiple parameters (47–49). A detailed description of the examined models is provided in Dataset S2 in the supplemental material. The final model selected was a combination of the models described by Talarida (38) and Machado and Robinson (46):

$$E_{PM}=E_{\max\;,P}\frac{C_{PM}^{\gamma_{P}}}{C_{PM}^{\gamma_{P}}+{\text{EC}}_{50,P}^{\gamma_{P}}},$$

where

(2) $$C_{PM}={\left(C_{P}^{\alpha }1_{W_{P}}(a)+C_{eq,M}^{\alpha }1_{W_{M}}(a)\right)}^{\frac{1}{\alpha }},$$

and

$$C_{eq,M}=E_{P}^{-1}\left(E_{M}\left(C_{M}\right)\right),$$

where *E_M_* is the killing effect of MQ and *E*_*p*_^−1^ is the inverse of the killing effect of PPQ, given by:

$$E_{P}^{-1}\left(x\right)={\text{EC}}_{50,P}{\left(\frac{x}{E_{\max\;,P}-x}\right)}^{\frac{1}{\gamma_{P}}},$$

where *E*_max,*P*_ and EC_50,*P*_ are the maximum killing effect of PPQ and the concentration at which half of the maximum killing effect is produced, respectively, and *W_P_* and *W_M_* are the killing windows of PPQ and MQ, respectively. Zero interaction is produced by equation 2 when α = 1; the values of 1 < α < ∞ and 0 < α < 1 produce antagonism and synergism, respectively. Note that PPQ is considered to be more potent than MQ (see Dataset S2 in the supplemental material for further information).

**FIG 5:**
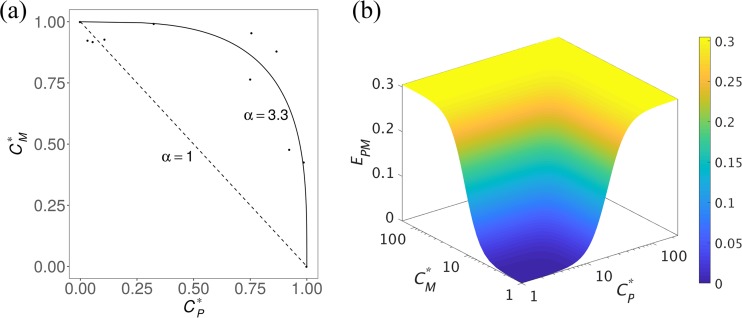
Interaction between PPQ and MQ and their combined effect. (a) Isobologram presented in Davis et al. (13 [adapted with permission of the authors]) showing a strong antagonistic interaction between PPQ and MQ. The dashed and solid lines show the zero-interaction isobole and our fitted curve to the data points (estimated PPQ-MQ interaction parameter is α = 3.3), respectively. C_M_* = *C_M_*/EC_50,*M*_ and C_P_* = *C_M_*/EC_50,*P*_ are the normalized concentrations of MQ and PPQ, respectively. (b) Combined effect of PPQ and MQ, i.e., *E_PM_* (the C_M_* and C_P_* axes are log scaled), when PPQ-MQ interaction parameter (α) equals 3.3. The maximum killing effects and sigmoidicity of PPQ and MQ are considered equal (i.e., *E*_max,*P*_ = *E*_max,*M*_ = 0.3 and γ_*P*_ = γ_*M*_ = 3) throughout the model fitting to conform with the data provided by Davis et al. 2006 (13).

Isobolograms, widely used in pharmacology and toxicology studies, can inform on the nature of drug-drug interactions. These present data on the parasiticidal effect of paired drug concentrations. The combination of drug concentrations is then compared with the zero-interaction isobole (also known as linear isobole) (50) (see Fig. 5a). When the pairs of drugs concentrations are close to the linear isobole, zero-interaction is inferred, and when they lie significantly above or below the linear isobole, antagonism or synergism, respectively, can be inferred.

Using this approach, Davis et al. (13) showed that the paired PPQ and MQ data were significantly above the zero-interaction isobole (dashed line), indicating a strong antagonistic interaction between PPQ and MQ (Fig. 5a). The combined killing effect of PPQ-MQ, *E_PM_*, was fitted to these data, and the PPQ-MQ interaction parameter was estimated to be α = 3.3. Figure 5b shows the predicted *E_PM_* for α = 3.3 for various PPQ and MQ concentrations. The killing effects of DHA and PPQ-MQ were applied to equation 1 to estimate the combined effect of DHA-PPQ-MQ and simulate the PD model (see Dataset S2 in the supplemental material for further details).

### Model simulation.

Latin Hypercube Sampling (LHS) was used to efficiently sample the parameter space (51) and simulate the PK profiles and parasitological responses. The distributions of the parameter values of the PK and PD models are presented in Tables 2 and 3, respectively. A triangular distribution was used for generating samples of α, with a peak at α = 3.3, estimated by fitting the model to the data, as explained in the previous section. The lower and upper bounds were selected to be 1 (zero interaction) and 10 (very strong antagonism), respectively. The initial parasite burden was assumed to have a log-normal distribution with a geometric mean of 1.14 × 10^11^ and a standard deviation of 1.13 on a log scale. The corresponding 5th and 95th percentiles of parasitemia are 1.78 × 10^10^ and 7.2 × 10^11^, respectively (Table 3).

**TABLE 3:** Statistical distribution of the initial parasite burden and parameter values of the PD model^*a*^

Parameter	Drug	Distribution	Description
*N*_0_		log*N*(25.46,1.13)	Initial no. of parasites
μ_0_		*DU*(4,16)	Mean of initial parasites age distribution (h)
σ_0_		*DU*(2,8)	SD of initial age distribution (h)
PMF		TRI(8,12,10)	Parasite multiplication factor (/48-h cycle)
*E*_max_^*b*^	DHA	TRI(0.49,0.69,0.59)	Maximum killing effect
	PPQ	TRI(0.19,0.50,0.35)	
	MQ	TRI(0.09,0.43,0.26)	
EC_50_ (ng/ml)^*c*^	DHA	*U*(1.44,532.05)	Concn producing *E*_max_/2 effect
	PPQ	*U*(11.56,94.19)	
	MQ	*U*(20.48,1087.22)	
γ	DHA	log*N*(1.31,0.65)	Sigmoidicity of the concn-effect curves
	PPQ	log*N*(1.35,0.66)	
	MQ	log*N*(0.97,0.54)	
α	PPQ-MQ	TRI(1,10,3.3)	Interaction parameter

a Terms: TRI(*l*, *h*, *m*), triangular distribution with peak at *m*, lower limit of *l*, and higher limit of *h*; DU(*l*, *h*), discrete uniform distribution with lower and higher limits *l* and *h*, respectively; *U*(*l*, *h*), continuous uniform distribution with lower and higher limits *l* and *h*, respectively; log*N*(μ, σ), log-normal distribution derived from a normal distribution with the mean μ and standard deviation σ. The killing windows of the drugs were as follows (17): *W_D_* = [6 44], *W_P_* = [12 36], and *W_M_* = [18 40].

b See Dataset S3 in the supplemental material for further details.

c The lower limit of the distribution of EC_50_ was chosen to be the *in vitro* IC_50_ (the concentration that inhibits the growth of parasites by 50%) of free drug, obtained by adjusting for the *in vitro* drug bindings. The higher limit was chosen to be half of the maximum drug concentration of the median of the PK profiles (17).

The probability of cure (i.e., 1 − the probability of failure) was used as a measure of drug efficacy, and Kaplan-Meier survival analysis was carried out on simulated parasite versus time profiles of the patients to estimate the probabilities of cure at day 42 of follow-up. Treatment failure was defined as parasite recrudescence, in which the peripheral parasitemia exceeded the microscopic limit of detection (50 parasites/μl or a total parasite biomass of 2.5 × 10^8^).

Dosing regimens recommended by the WHO were used in the simulations. These included 18.0 mg/kg/day for PPQ and 4.0 mg/kg/day for DHA for 3 days. Current guidelines recommend a total dose of 25 mg/kg of MQ in combination with 4 mg/kg/day of artesunate (19). Splitting the dose of MQ (8.3 mg/kg/day for 3 days) improves the bioavailability of MQ, is better tolerated, and has a greater efficacy (52). Higher daily doses of MQ are associated with significant side effects (53), and thus modeling explored the minimum dosage of MQ that results in optimal efficacy. Hence, the administered dose of MQ was varied, and the corresponding TACT efficacy was estimated.

Different scenarios were considered, simulating resistance to DHA and/or PPQ. To simulate different degrees of PPQ and DHA resistance, EC_50_, *E*_max_, and *W* were varied over the limited sampling intervals of the range of values given in Table 3. Since EC_50_ is not measurable in experiments, a wide initial range was considered for it: between the concentration that inhibits the growth of parasites by 50% *in vitro*, IC_50_ (adjusted for protein binding), and half the maximum concentration of the median of the PK profiles (17). As shown in Fig. 3, the limited sampling intervals for this range are able to produce the probabilities of cure observed in the regions with different levels of parasite susceptibility to drugs.

## Supplementary Material

Supplemental file 1
