# Supplementary material for: Investigating the Efficacy of Triple Artemisinin-Based Combination Therapies for Treating Plasmodium falciparum Malaria Patients Using Mathematical Modeling
Source: Antimicrob Agents Chemother. 2018 Oct 24;62(11):e01068-18. doi: 10.1128/AAC.01068-18 (PMC6201091; doi:10.1128/AAC.01068-18)
Supplement: Supplemental file 1 [file zac011187578s1.pdf]

Supplementary Materials

1 Other manifestations of resistance

1.1 Maximum killing effect, E_{max}

Here we examine the probability of cure on day 42 of follow-up when E_{max} is the resistance manifestation. Fig. S1 shows the results when $E_{max,P}$ varies across the deciles of its sampling interval; samples are taken from uniform distributions over each decile. Similar to the results of Section "Artemisinin resistance", adding one dose of MQ to the ACT (blue curve) can increase the probability of cure, but is not sufficient. In order to reach the probability of cure of above 90% for all of the deciles, we need three doses of MQ. Of interest, the magnitude of the effect of resistance on probability of cure in this case is close to that of EC_{50}; resistance to DHA is also considered, *i.e.* $EC_{50,D} \in (50, 100]$.

1.2 Killing window, W

We now shorten the size of killing window, W, of PPQ for the intra-erythrocytic parasite life cycle, by increasing the lower limit of the W and fixing the higher limit. The results for the ACT show that shortening the killing window can significantly reduce the probability of cure, but again, adding MQ to the compound can pull up the probabilities of cure. To achieve a probability of cure of at least 90%, three 8.3 mg/kg doses of MQ are required.

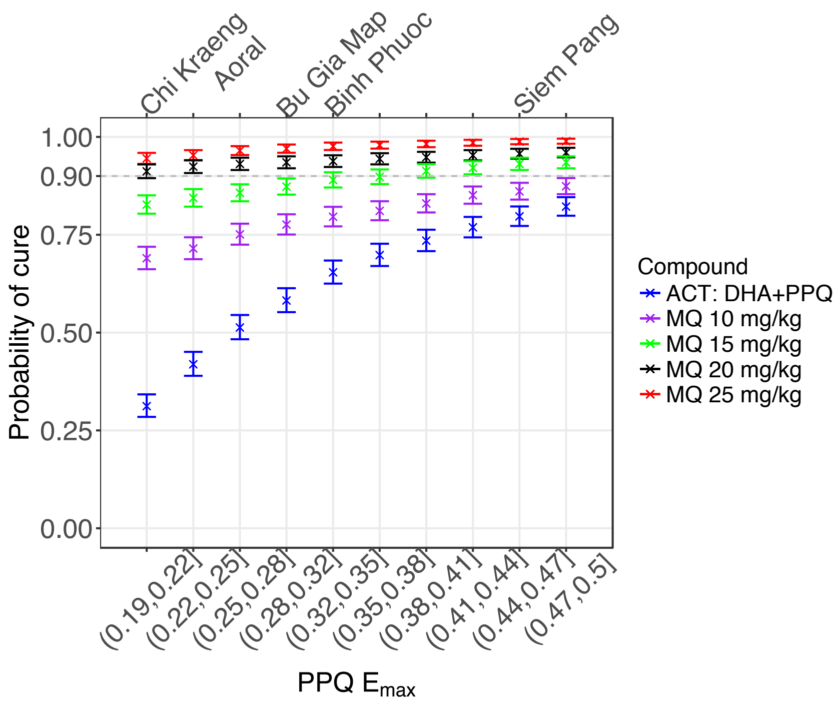

Figure S1: **The probability of cure on day 42 of follow-up when E_{max} of PPQ varies over the deciles of** $(0.19, 0.50]$**.**

Dosing regimens of PPQ and DHA are 18.0 mg/kg and 4.0 mg/kg, respectively, on days 1, 2 and 3. Purple: 10 mg/kg (3.3 mg/kg/day for three days) dose of MQ is added. Green: 15 mg/kg (5 mg/kg/day for three days) dose of MQ is added. Black: 20 mg/kg (6.7 mg/kg/day for three days) dose of MQ is added. Red: 25 mg/kg (8.3 mg/kg/day for three days) dose of MQ is added. The top labels show the geographical regions in South-East Asia (Table 1) that have observed DHA-PPQ cure rates equal to the corresponding simulated values. Error bars show the 95% confidence intervals of Kaplan-Meier analysis.

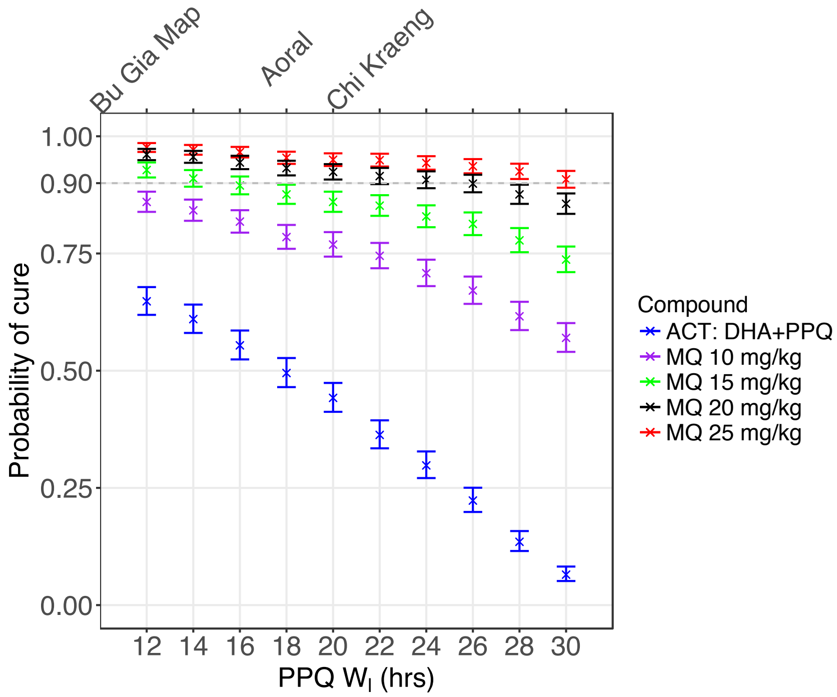

Figure S2: **The probability of cure at day 42 of follow-up when the size of the parasite killing window (W) for PPQ is reduced by increasing the lower limit, W_l, from 12 to 30.**

The higher limit, W_u, is constant and equal to 36 hours. Purple: 10 mg/kg (3.3 mg/kg/day for three days) dose of MQ is added. Green: 15 mg/kg (5 mg/kg/day for three days) dose of MQ is added. Black: 20 mg/kg (6.7 mg/kg/day for three days) dose of MQ is added. Red: 25 mg/kg (8.3 mg/kg/day for three days) dose of MQ is added. The top labels show the geographical regions in South-East Asia (Table 1) that have observed DHA-PPQ cure rates equal to the corresponding simulated values. Error bars show the 95% confidence intervals of Kaplan-Meier analysis.

2 Modelling combined killing effect

2.1 Models of drug interaction

There are two prominent empirical approaches for modelling zero-interaction: *Loewe additivity* (1) and *Bliss independence* (2). Loewe additivity is based on the idea that two non-interacting drugs differ only in their potency, and was originally formulated as

$$1 = \frac{C_1}{c_1} + \frac{C_2}{c_2}, \tag{2.1}$$

where c_1 and c_2 are the concentrations of drugs 1 and 2, respectively, that each individually (*i.e.* not in combination) produces a specified effect E_{12}, and C_1 and C_2 are the drug concentrations in a combination that together produce E_{12} — for brevity, the formulae are defined for two drugs, but they can be readily extended for multiple drugs. Eqn. (2.1) is known as a *linear isobole*, which is widely used in pharmacology and toxicology as a reference to identify drug interactions. Loewe first put forward this model, which was then investigated more rigorously by Berenbaum (1985) and others.

Loewe additivity is suggested to be a suitable concept for zero-interaction when the combined drugs have similar modes of action (4, 5). However, when the drugs are believed to act independently, Bliss independence is more appropriate. This model is based on a probabilistic perspective, defined as

$$E_{12} = E_1 + E_2 - E_1 E_2 \tag{2.2}$$

where E_1 and E_2 are the individually produced effects by drugs 1 and 2, respectively.

Ultimately, deviations from a selected zero-interaction reference model would determine the degree of synergistic/antagonistic interaction in certain drug combinations. Note that despite the fundamental differences of Loewe additivity and Bliss independence, it has been shown that they indicate the same nature of drug interactions in the majority of cases (6).

2.2 Combined effect of DHA-PPQ-MQ

Statistical models can be used to define E_{PM}, *e.g.* Carter et al. (1988) used a generalised linear model with the logit link function:

$$\log\left(\frac{E_{PM}}{1-E_{PM}}\right) = \beta_0 + \beta_1 C_P + \beta_2 C_M + \beta_3 C_P C_M,$$

where C_P and C_M are the concentrations of PPQ and MQ, respectively, and $\beta_0, \ldots, \beta_3$ are the coefficients of the model. Similar statistical models can be found in (8, 9).

Another set of models include only one parameter to incorporate the effect of interaction (4, 10, 5). These models are more specified to the framework of drug interaction, in contrast to the statistical models. Here, we focus on the models with one parameter of interaction — noting that statistical models are shown to be readily transformable to these models, *e.g.* see (7).

One of the most frequently used models to describe the combined effect is Greco's

model (4), defined by

$$
\begin{aligned}
1 = & \frac{C_P}{EC_{50,P}\left(\frac{E_{PM}}{E_{max,P}-E_{PM}}\right)^{\frac{1}{\gamma_P}}} + \frac{C_M}{EC_{50,M}\left(\frac{E_{PM}}{E_{max,M}-E_{PM}}\right)^{\frac{1}{\gamma_M}}} \\
& + \frac{\alpha C_P C_M}{EC_{50,P}EC_{50,M}\left(\frac{E_{PM}}{E_{max,P}-E_{PM}}\right)^{\frac{1}{2\gamma_P}}\left(\frac{E_{PM}}{E_{max,M}-E_{PM}}\right)^{\frac{1}{2\gamma_M}}}
\end{aligned} \tag{2.3}
$$

where the subscripts P and M denote which drug the parameters correspond to. The interaction parameter, α, incorporates the influence of the interaction between the drugs, where, for Eqn. (2.3), $\alpha = 0$, $-1 < \alpha < 0$ and $\alpha > 0$ produce zero-interaction, antagonism and synergism, respectively. Note that we should have $E_{PM} < E_{max,P}$ and $E_{PM} < E_{max,M}$, otherwise, Eqn. (2.3) would not yield a real-valued solution for E_{PM}. These conditions thus limit the utility of Greco's model to cases where $E_{max,P} \neq E_{max,M}$.

Tallarida (2006) put forward a broader framework based on the Loewe additivity, from which Greco's model can be derived as a special case. In addition, it overcomes the aforementioned limitation on the values of E_{PM}. In Tallarida's approach, we first identify the more potent drug, say PPQ; this can be done by carrying out *in vitro* susceptibility tests or comparing the parasite reduction ratios derived from clinical efficacy studies. Then, we find the concentration of PPQ that is equally effective as MQ at concentration C_M, using

$$C_{eq,M} = E_P^{-1}\left(E_M(C_M)\right),$$

where E_P^{-1} is the inverse function of E_P, given by

$$E_P^{-1}(x) = EC_{50,P}\left(\frac{x}{E_{max,P}-x}\right)^{\frac{1}{\gamma_P}},$$

Then, the zero-interaction model is obtained via

$$E_{PM} = E_{max,P} \frac{C_{PM}^{\gamma_P}}{C_{PM}^{\gamma_P} + EC_{50,P}^{\gamma_P}},$$

where

$$C_{PM} = C_P \mathbf{1}_{W_P}(a) + C_{eq,M} \mathbf{1}_{W_M}(a). \tag{2.4}$$

Subsequently, Eqn. (2.4) can be modified to accommodate an interaction between drugs. For example, Tallarida (2000) suggests changing this equation to C_{PM}/α, where α is the interaction parameter. However, we dismiss this method as it does not produce the observed antagonistic isoboles (see Fig. 5), hence, it will not provide a good fit to data. In order to obtain a form of E_{PM} similar to Greco's model, Eqn. (2.3), we then modified Eqn. (2.4) to incorporate the effect of an interaction between drugs. Adding $\alpha C_P C_{eq,M}$ as an extra term to this equation provides a good fit to the data for $\alpha = -0.132$, but, the resultant E_{PM} is non-monotonic, which is biologically infeasible. We also tried other terms such as $\alpha\sqrt{C_P C_{eq,M}}$, but they similarly failed to give either a good fit or a monotonic effect. Hence, the models of form Eqn. (2.3) did not produce an appropriate E_{PM}, as also outlined by White et al. (2003) and Machado, Robinson (1994).

We then turned to using the model introduced by Machado, Robinson (1994):

$$C_{PM} = \left(C_P^{\alpha}\ \mathbf{1}_{W_P}(a) + C_{eq,M}^{\alpha}\ \mathbf{1}_{W_M}(a)\right)^{\frac{1}{\alpha}},$$

where zero-interaction is produced when $\alpha = 1$. The values of $1 < \alpha < \infty$ and $0 < \alpha < 1$ produce antagonism and synergism, respectively. The model provides a good fit to the

data (see Fig. 5a), and importantly, a biologically feasible killing effect, E_{PM} (see Fig. 5b). Therefore, we selected this model for E_{PM}, and used it in the combined effect, Eqn. (1), of the TACT.

To conform with the data provided by Davis et al. (2006) (13), the maximum killing effects and sigmoidicity of PPQ and MQ are considered equal (*i.e.* $E_{max,P} = E_{max,M} = 0.3$ and $\gamma_P = \gamma_M = 3$) throughout the model fitting. However, the considered range of variation for α in the simulations is significantly larger than the potential variations due to $E_{max,P} \neq E_{max,M}$ and/or $\gamma_P \neq \gamma_M$, hence, these assumptions do not invalidate the results (see Table 3).

3 Calculating E_{max} using the parasite reduction ratio (PRR)

We are interested in finding how E_{max} is related to the parasite reduction ratio (PRR). We can estimate PRR by

$$PRR = \frac{N_0}{\sum_{a=1}^{48} N(a, t_0 + T)}$$

where T is the time when we count the number of parasites (*e.g.* $T = 48$ hrs) to calculate PRR, and N_0 is the initial number of parasites at time t_0. Then, we have

$$\sum_{a=1}^{48} N(a, t_0) \prod_{\tau=0}^{T-1} (1 - E(a_\tau, t_0 + \tau)) = \frac{N_0}{\text{PMF} \times \text{PRR}},$$

where $a_\tau = [(a+\tau) \bmod 48]$. Thus, we use numerical methods to solve the above equation for E_{max}. The estimated E_{max} values are listed in Table 3. Note that it is extremely important to take account of the details of the clinical efficacy studies, by which the PRRs of the drugs are obtained. We used the following PRRs and the dosing regimens to estimate E_{max} for each drug:

- $\text{PRR}_{DHA} = 10^4$: seven 2 mg/kg doses of DHA are administered (14).
- $\text{PRR}_{PPQ} = 2951$: one 14.1 mg/kg dose of PPQ is administered (15).
- $\text{PRR}_{MQ} = 100$: one 25 mg/kg dose of MQ is administered (14).

The obtained E_{max} is then used as the median of the triangular distribution (see Table 3). The lower ($E_{max,l}$) and higher ($E_{max,h}$) limits of the distribution are assumed that correspond to 50-fold increase and decrease in the above PRRs, respectively, which yields

$$E_{max,l} = E_{max} - \frac{\log(50)}{||W||},$$
$$E_{max,h} = E_{max} + \frac{\log(50)}{||W||},$$

where $||W||$ is the size of killing window of the drug (16).

References

1. Loewe S. 1928. Die quantitativen probleme der pharmakologie. Ergebnisse der Physiol 27:47–187.

2. Bliss CI. 1939. The toxicity of poisons applied jointly. Ann Appl Biol 26:585–615.

3. Berenbaum MC. 1985. The expected effect of a combination of agents: the general solution. J Theor Biol 114:413–431.

4. Greco WR, Bravo G, Parsons JC. 1995. The search for synergy: a critical review from a response surface perspective. Pharmacol Rev 47.

5. Tallarida RJ. 2006. An overview of drug combination analysis with isobolograms. J Pharmacol Exp Ther 319.

6. Drusano GL, D'Argenio DZ, Symonds W, Bilello PA, McDowell J, Sadler B, Bye A, Bilello JA. 1998. Nucleoside analog 1592u89 and human immunodeficiency virus protease inhibitor 141w94 are synergistic *in vitro*. Antimicrob Agents Chemother 42:2153–2159.

7. Carter WH, Gennings C, Staniswalis JG, Campbell ED, White KL. 1988. A statistical approach to the construction and analysis of isobolograms. J Am Coll Toxicol 7:963–973.

8. Gennings C. 2000. On testing for drug/chemical interactions: definitions and inference. J Biopharm Stat 10:457–467.

9. Plummer JL, Short TG. 1990. Statistical modeling of the effects of drug combinations. J Pharmacol Methods 23:297–309.

10. Machado SG, Robinson GA. 1994. A direct, general approach based on isobolograms for assessing the joint action of drugs in pre-clinical experiments. Stat Med 13:2289–2309.

11. Tallarida RJ. 2000. Drug synergism and dose-effect data analysis. Chapman & Hall/CRC.

12. White D, Slocum H, Brun Y, Wrzosek C, Greco W. 2003. A new nonlinear mixture response surface paradigm for the study of synergism: a three drug example. Curr Drug Metab 4:399–409.

13. Davis TME, Hamzah J, Ilett KF, Karunajeewa HA, Reeder JC, Batty KT, Hackett S, Barrett PHR. 2006. *In vitro* interactions between piperaquine, dihydroartemisinin, and other conventional and novel antimalarial drugs. Antimicrob Agents Chemother 50:2883–2885.

14. White NJ. 1997. Assessment of the pharmacodynamic properties of antimalarial drugs *in vivo*. Antimicrob Agents Chemother 41:1413–1422.

15. Pasay CJ, Rockett R, Sekuloski S, Griffin P, Marquart L, Peatey C, Wang CYT, O'Rourke P, Elliott S, Baker M, Möhrle JJ, McCarthy JS. 2016. Piperaquine monotherapy of drug-susceptible *Plasmodium falciparum* infection results in rapid clearance of parasitemia but is followed by the appearance of gametocytemia. J Infect Dis 214:105–113.

16. Zaloumis S, Humberstone A, Charman SA, Price RN, Moehrle J, Gamo-Benito J, McCaw J, Jamsen KM, Smith K, Simpson JA. 2012. Assessing the utility of an anti-malarial pharmacokinetic-pharmacodynamic model for aiding drug clinical development. Malar J 11:303.
